# Touching Base: support group for Individuals living with a severe and enduring eating disorder

**DOI:** 10.1186/2050-2974-2-S1-O61

**Published:** 2014-11-24

**Authors:** Heather Litchfield

**Affiliations:** 1 Eating Disorder Unit, North Western Mental Health, The Royal Melbourne Hospital, Melbourne, Australia

## 

Touching Base was developed as there was a lack in services for patients that have been living with their illness for twenty plus years. These patients have had many admissions, tried multiple services and overtime have learned to live with their eating disorder but as a consequence and have become withdrawn from their communities and isolated from family and friends.

Beginning this group in the start of 2013 it was a challenge to engage these patients, alterations to the group structure needed to be made to accommodate participants in this new initiative such as making the group open, changing the duration of the group and time of day the group occurred.

Touching Base is now a thriving group where participants bring topics for discussion, encouraging, supporting and relating to one and other, developing positive friendships. Making this group available also gives staff the opportunity to engage participants in other areas of treatment such as linking participants in with outpatient supports. Touching Base continues to acknowledge individuals lived experiences with no judgements and no expectations, giving participants the opportunity to share their experiences and create positive friendship groups.

This abstract was presented in the **Peer Support** stream of the 2014 ANZAED Conference.

